# Involving young people in sexual health research and service improvement: conceptual analysis of patient and public involvement (PPI) in three projects

**DOI:** 10.1136/bmjsrh-2022-201611

**Published:** 2022-10-28

**Authors:** Ruth Lewis, Nicola Boydell, Carolyn Blake, Zoe Clarke, Kirsten Kernaghan, Christina McMellon

**Affiliations:** 1 MRC/CSO Social and Public Health Sciences Unit, University of Glasgow, Glasgow, UK; 2 Centre for Biomedicine Self and Society, Usher Institute, University of Edinburgh, Edinburgh, UK; 3 Health Opportunities Team, Edinburgh, UK; 4 Office for Health Improvement and Disparities, London, UK; 5 Chalmers Centre, NHS Lothian, Edinburgh, UK; 6 Moray House School of Education and Sport, University of Edinburgh, Edinburgh, UK

**Keywords:** health services research, patient participation, sexual health, adolescent

## Abstract

**Background:**

Although increasingly recognised as valuable within sexual and reproductive health (SRH) research and service improvement, examples of patient and public involvement (PPI) are underdocumented, including specific issues relating to young people’s involvement. This article aims to contribute to greater transparency about the practical, methodological and ethical considerations of SRH-related PPI with young people, and to offer recommendations for their meaningful involvement.

**Methods:**

Guided by a conceptual tool for evaluating youth participation (the ‘7P’ framework), we analysed learning from PPI within three projects (two academic studies and one service improvement project) that worked *with* young people to shape sexual health research and practice in Scotland.

**Analysis:**

Cross-project analysis of seven interconnected domains (purpose, positioning, perspectives, power relations, protection, place and process) generated productive dialogue about the nuances of meaningfully involving young people in shaping SRH research and services. Key learning includes the importance of: young people’s early involvement in agenda-setting for SRH improvement; developing trusting partnerships that can support involvement of diverse groups of young people; creating multiple ways for young people to contribute, including those that do not rely on direct conversation; and formative evaluation of young people’s experiences of involvement.

**Conclusions:**

Mainstreaming young people’s meaningful involvement in shaping SRH research and services requires systems-level change. Resources are required to support SRH researchers and practitioners to share learning and build sustainable multi-sector partnerships, which in turn can increase opportunities for young people from diverse groups to engage with SRH-related PPI activities.

**WHAT IS ALREADY KNOWN ON THIS TOPIC**

- Patient and public involvement (PPI) is widely recognised as important for improving sexual and reproductive health (SRH) research and services, but there is a lack of clarity about what meaningful SRH-related PPI looks like, especially when working with young people.

**WHAT THIS STUDY ADDS**

- Frameworks of youth participation can provide useful ‘thinking tools’ for planning and evaluating young people’s involvement in PPI activities, and reflecting on interconnections between practical, methodological and ethical considerations.

**HOW THIS STUDY MIGHT AFFECT RESEARCH, PRACTICE OR POLICY**

- Increasing young people’s meaningful involvement within SRH-related PPI requires more opportunities and resources to enable practitioners, researchers and the wider SRH community to discuss challenges, share good practice, and collaborate.

## Introduction

Involving young people in shaping sexual and reproductive health (SRH) research and services is posited as a way to improve service access and uptake, and fulfil young people’s right to influence policies that affect them (“nothing about us without us”).^1 2^ Collaboration between SRH service users, providers and researchers is not new, but rather builds on long histories of activism between different communities, allies and health practitioners to progress healthcare experiences and rights relating to, for example, contraception, abortion and HIV/AIDS.^3 4^ These “grassroots” collaborations increasingly sit alongside a shift within contemporary UK healthcare services and research towards a more formalised language of, and practices associated with, patient and public involvement (PPI) (see box 1). Major health research funders now routinely expect some element of PPI, and various standards and reporting guidelines aim to support researchers with PPI endeavours.^5–7^ Yet, despite offering exciting opportunities to democratise health improvement, some PPI activities are critiqued for being tokenistic.^8 9^

Box 1 **Involving young people in sexual and reproductive health (SRH) improvement – a note on terminology**

Throughout this article, we use the term ‘patient and public involvement’ (PPI) when referring to young people’s involvement in shaping sexual health research and practice in three projects (two academic research studies and one NHS service improvement project). We recognise that the term PPI, although widely used in the UK at present, is not underpinned by a universally agreed definition or set of practices. Alongside PPI, a variety of other terms such as ‘participation’, ‘engagement’, ‘co-design’ and ‘co-production’ are often used synonymously, yet conceptualised variably in health sciences and allied fields. This poses challenges for communicating across disciplines, sectors of practice and national contexts. Debates around terminology relating to practices of involvement are beyond the scope of this article, which aims to critically reflect upon young people’s involvement in shaping sexual and reproductive health research and practice.

For further critical discussion of participatory practices (broadly defined) see Palmer on the ‘participatory Zeitgeist’,^28^ Fransman on engagement,^29^ Williams and colleagues on distinctions between co-production and PPI^9^ and Redman and colleagues on co-production of knowledge.^30^

Within SRH, some argue that there is lack of clarity about what good PPI “looks like”.^10^ In part, this may stem from limited advice about how to translate general PPI guidance into appropriate practices within SRH, where stigma and need for privacy may mitigate against visible involvement.^11–13^ Particularly remarkable is lack of attention to the specific challenges of involving young people (hereafter referred to as YP) in SRH-related PPI – a surprising absence given that this age group continues to experience a high burden of poor sexual health outcomes,^14 15^ and may be especially susceptible to power differentials within PPI. Opportunities to advance practice are further constrained by limited publication; despite initiatives to increase the visibility of SRH-related PPI,^16^ the category for submission of articles focused on involvement often remains unclear, and likely only a fraction of PPI is “written up”.^17^ Among published work, a revelatory audit of PPI within UK SRH services and research illuminated various challenges including: reports of innovation being undermined by standardised NHS PPI systems; lack of identified PPI goals; conflation of PPI and qualitative research; limited ‘patient satisfaction’ approaches, and poor resourcing.^10^

In this context of insufficient practical guidance and dialogue, conceptual frameworks of youth participation potentially offer valuable tools to advance YP’s involvement in shaping SRH research and services. Among various models, one of the most influential is Hart’s^18^ “ladder of participation” which characterises levels of participation according to degrees of power-sharing between adults and YP. While this model draws much-needed attention to power dynamics, it has been critiqued for implying that youth-initiated participation is inherently superior to adult-initiated participation that works to share decision-making with YP.^19^ Moving away from this hierarchical view, Cahill and Dadvand propose an alternative, the 7P model^20^ (see online supplemental file), which provides seven domains – purpose, positioning, perspectives, power relations, protection, place and process – as a series of “thinking tools” to aid planning and evaluation of youth participation. This framework emphasises the *inter-connectedness* of actions across these domains, and the dynamic nature of participatory processes.

10.1136/bmjsrh-2022-201611.supp1 Supplementary data

In this article, we apply the 7P framework to critically reflect on PPI within three projects (one service improvement project,^21^ two university-led research studies^22 23^) that worked *with* YP to shape research and services. In so doing, we aim to contribute to increased transparency and dialogue about key considerations and challenges (practical, methodological and ethical) relating to SRH-related PPI.

## Methods

Details of PPI elements for each project are presented in table 1. All three projects were conducted in Scotland between 2018 and 2020 and and were subject to research governance and/or ethics review. With the exception of NB, all authors were members of one or more project teams.

**Table 1:** Background details of three sexual and reproductive health projects involving patient and public involvement (PPI) with young people

Project name	Project 1: Improving Care-Experienced Young People’s (CEYP) Access to SRH Services in Edinburgh	Project 2: CONUNDRUM (Condom & Contraception Understandings: Researching Uptake and Motivations)	Project 3: Communicating Sexual Consent
Duration	5 months	14 months	8 months
Location	Edinburgh and Lothians	Scotland-wide	Greater Glasgow & Clyde, Lothians, Lanarkshire
Sector/s of team	Collaboration between NHS sexual health staff (KK and colleagues) and a youth organisation (ZC)	Led by university-based researchers (RL, CB, CM and colleagues), in collaboration with sexual health leads from Scottish NHS health boards and Scottish Government	University-based researchers (CM and colleague)
How did the project arise?	Conceived by clinic-based sexual health practitioners and community-based health promotion team in response to team reflection on need to improve CEYP’s access to, and experiences of, SRH services. Funded by Edinburgh Family Planning Trust and NHS Lothian.	A need for research evidence was identified and funded by three NHS Scotland health boards, in partnership with Scottish Government, in response to changes in YP’s use of free condom services and some forms of LARC. Research team engaged through competitive tender process. Research questions developed through PPI activities with stakeholders, including YP.	Conceived and funded by three NHS Scotland health boards, in partnership with Scottish Government, in response to identified need for resources on sexual consent. Research team engaged through competitive tender process.
PPI-related project aims	To involve care-experienced YP in identifying key priorities for service improvement within a specified SRH clinic, including co-developing an effective referral pathway.	To involve YP in co-developing a mixed methods study exploring factors shaping condom and contraception use among YP in Scotland.	To ensure that YP’s views inform the development of a health promotion film being developed by NHS health boards in Scotland to promote communication around sexual consent.
Methods used to involve young people in PPI activities	Multi-phase process with 33 YP aged 13–25 years, including: *Exploring CEYP’s experiences* of SRH services via interviews (n=15); *Establishing YP’s priorities* via consultation activities designed to establish which issues identified in interviews were the most important to action (n=12); *Co-developing recommendations for service improvement* via a workshop with 6 CEYP and SRH clinic staff. Workshop focused on identifying action points for SRH service improvement for CEYP.	Multi-phase process with 60 YP aged 16–24 years, including: *Co-developing research priorities and questions* via six in-person workshops with 38 YP. Creative drawing methods and discussion activities used to: (1) explore views on factors shaping condom and contraception use; (2) identify priority research questions; (3) generate ideas for survey items addressing research questions; *Co-developing online survey* via two virtual meetings with 9 YP to review and refine: draft survey items; the online format; information provided to potential respondents; strategy for advertising survey via social media; *Co-developing policy recommendations* via two virtual meetings with 13 YP. Meetings included review and discussion of study findings; activities using virtual notepads to collaboratively generate and discuss ideas for policy change.	Multi-phase process with 58 YP aged 16–25 years, including: *Co-developing and piloting research tools* (eg, topic guides) via two meetings with 5 YP. Meetings focused on ensuring language and questions were relevant, interesting, and comprehensible for YP; *Exploring YP’s views* via focus groups and individual interviews; *Collaborative data interpretation, and co-developing a YP’s report about the study* via five meetings with 3 YP. Meetings focused on ‘sense-checking’ study findings with YP, exploring their ideas about what the findings mean for the consent resource that was developed, and co-developing the content, structure and graphics for the YP’s report.
How were young people recruited for PPI activities?	Multi-strand approach including recruitment via: (1) third sector youth services; (2) secure services and (3) Young People’s Centres.	Multi-strand approach including recruitment via: (1) a diverse range of youth groups and third sector youth organisations and (2) research team’s existing contacts with youth workers.	Multi-strand approach including recruitment via: (1) youth organisations and (2) research team’s existing contacts with YP involved in previous sexual health-related research.
Were young people compensated for their involvement?	YP were not compensated for their participation in consultation or workshop activities. YP who participated in interviews received a £5 voucher for their involvement.	Yes – YP received vouchers for participation in workshops and meetings (approx. £25 per hour, with no preparation time required).	Yes – both youth advisors and research participants received vouchers for their participation (approx. £20 per hour).

CEYP, care-experienced young people; LARC, long-acting reversible contraception; NHS, National Health Service; SRH, sexual and reproductive health; YP, young people.

Our analytic process was multistage and iterative. Each project team used the 7P framework to assess strengths and tensions relating to each domain within their project, followed by discussion among all authors to further probe the strengths and tensions identified (see table 2). We then compared issues encountered in each domain *across* the different projects, and interactions between the domains. Collective discussion occurred during nine virtual analytic meetings. Our team’s multi-sector composition (eg, backgrounds in nursing, public health, youth work, medical sociology) enabled us to compare interpretations and learning points from different disciplinary perspectives.

**Table 2:** Involving young people in sexual and reproductive health (SRH)-related patient and public involvement (PPI): example insights generated through using the 7P framework as a reflective tool

Example questions reflected on per 7P domain	Project 1: Improving Care-Experienced Young People’s Access to SRH Services in Edinburgh	Project 2: CONUNDRUM (Condom & Contraception Understandings: Researching Uptake & Motivations)	Project 3: Communicating Sexual Consent
**PURPOSE** *What opportunities are constructed to enable young people to play an active role in shaping or evolving project objectives*?	The ‘problem’ that sparked the initial project idea was identified by clinic- and community-based SRH practitioners based on their experiences of working with care-experienced young people (CEYP) and carers. The project was then designed by SRH researchers and practitioners, who sought research funding. The project team developed the project objectives to redesign care pathways and improve CEYP’s access to SRH services. They sought to centre CEYP as project collaborators, but YP were notactively involved as co-creators of the project vision.	YP were involved in shaping study objectives (eg, via workshops to define the ‘problem’ and identify priority questions around which to build evidence). Differences arose between YP’s views that barriers to using SRH services should be a focus of study, and study commissioners’ initial views that these were already well understood so study resources were better directed to exploring wider social influences on condom and contraception use. These differing views on the purpose of the research were discussed with commissioners and the study priorities were reworked to include a focus on services, but more could have been done to communicate with YP about how their input shaped the study purpose (ie, **Process**).	While the project was driven by recognition that YP needed to be involved in shaping resources designed for YP about sexual consent, the project objective (to contribute to development of a short film) was set by study commissioners from the beginning. Although study commissioners demonstrated some flexibility around the project objective at the final research project meeting, opportunities for young advisors to shape the chosen approach to promoting consent were limited by pre-existing institutional funding agreements for the 'output'.
**POSITIONING** *How are young people positioned within the project and wider cultural discourses, and how might this limit what is initially imagined to be possible?*	Within UK policy discourse, CEYP are commonly positioned as both “seldom-heard” and “vulnerable”. While aware of reasons for these framings, the project team sought to (re)position CEYP as “active partners” in redesigning access to SRH services. They also wanted to move away from positioning CEYP as “research participants” where their perspectives would be ‘filtered’ through researchers, instead using participatory methods and activities (**Process**) to create opportunities for direct dialogue between practitioners and YP. However, NHS research management and governance around the need for safeguards (**Protection**) when working with CEYP implicitly shaped what was imagined to be possible within the project and limited the extent to which YP could shape the methods used (**Process**).	The project team’s positioning of YP as having valuable contributions to make included well-intentioned labelling of their role as “advisors” and “collaborators”. In reality, however, YP’s contributions were on a spectrum of involvement (eg, from one-off to more sustained inputs) that did not always reflect the positioning labels imposed by the research team.	YP were viewed by project commissioners and the research team as having “insider” knowledge about YP’s communication about consent and engagement with social media meaning their involvement was seen as an integral and invaluable aspect of research design. Study commissioners’ initial doubts about the feasibility of generating meaningful involvement by YP in the short project timescale were quickly quelled by early input from young advisors on ways to improve the research tools and language used in focus groups/interviews.
**PERSPECTIVES** *Whose perspectives and voices are included, excluded or privileged in the project?*	The project team recognised the heterogeneity among CEYP, and devised processes to reach and include those with different experiences of care including kinship care, foster care, care leavers, and YP residing in Young People’s Centres and secure settings. Although the project successfully involved a diverse group of CEYP, the participatory activities used (**Process**) likely privileged the voices of those most able to share their views and experiences via in-person dialogue with professionals. CEYP who did not engage with health services were not involved, arguably extending a pattern of societal exclusion.	Researchers tried to address inequitable patterns of involvement in SRH PPI by holding a series of smaller workshops and meetings with YP (**Process**), rather than one big event, and approaching youth organisations that support YP with minority identities and experiences (eg, LGBTQ+youth, black and minority ethnic youth). Yet efforts to involve diverse groups of YP via these channels likely inadvertently placed pressure on youth organisations operating with limited funds and receiving multiple requests from research teams. Trusting relationships between research teams and youth organisations need to be built in a sustained and reciprocal way, and there are challenges of doing this in the scope of discrete projects with limited time and resources.	Researchers tried to involve young advisors with a range of experiences, sexualities and genders, but tight project timescales and budget meant that recruitment for youth advisors ended up being through existing contacts with YP with previous experience of research about sexual health/sexuality who could quickly engage with the project. Young advisors were mostly women, politicised, white and heterosexual. Partially successful efforts were made to include more diverse views in workshops and interviews, but young men and YP from black and minority ethnic backgrounds were underrepresented.
**POWER RELATIONS** *How were relationships managed to ensure equity and respect was enacted between all parties?*	The project team was attuned to the widespread **Positioning** of CEYP as passive recipients of care within health services and sought to enact respectful relationships through participatory activities that foregrounded CEYP as valued contributors to redesigning SRH care pathways. This informed group agreements around equitable communication and respect for the experience and perspectives of all participants during PPI activities. Although a collaborative working group between CEYP and staff was planned as a way to promote equity, this aspect of the project was not realised. Resource constraints and institutional hierarchies limited the extent to which the priorities identified could be taken forward.	Efforts to promote equity and respect between the project team and YP included: (i) foregrounding the value of YP’s ideas and input into shaping the study in meetings with other stakeholders; (ii) involving YP in public discussion about the study findings (eg, as panellists in the webinar to launch the final report); and (iii) prioritising reciprocity between the project team and YP through support for their own endeavours (eg, providing input on research skills to support youth-led initiatives with their own research). Despite these efforts, more equitable power relations were limited by institutional requirements to deliver pre-agreed outputs.	Given the sensitivity of the topic and the tight timescale of the project, young advisors were recruited who had strong existing relationships with the researchers and experience of working together on related topics. The young advisors recommended offering options for YP’s participation including making a distinction between workshops about the topic of sexual consent and individual interviews about personal experiences (**Process**). Offering options within the research process gave YP control over their participation and choice about when and how to share their ideas, opinions and experiences.
**PROTECTION** *What is the balance between practices used to promote protection and those used to enhance participation?*	The project team sought to apply a trauma-informed approach. This led them to consider practices that could promote feelings of safety while enhancing CEYP’s participation in discussions about SRH services, for example, (i) collectively agreeing the boundaries of group discussion; (ii) working with CEYP to identify “safe”, “youth-friendly” locations (**Place, Power Relations**) for group work; and (iii) support from trusted (adult) team members attuned to implicit **Power Relations**. CEYP were accustomed to talking in a boundaried way, likely because of their experiences of interacting with adults around safety and disclosures. Nevertheless, it is possible that research governance processes, including necessary safeguards when researching around sex and healthcare with YP, served to limit opportunities for YP to define what safety meant to them in this context.	The project team was keenly aware that involvement in shaping a study about condom and contraception use could be personally and socially risky for YP. Attempts to promote feelings of safety and privacy included collaboratively agreeing ground rules around disclosures in group discussion, and arranging separate workshops for YP and other stakeholder groups involved in shaping the project (eg, SRH practitioners and policymakers). Although YP appeared to value participating in their own spaces, it is possible that the project team’s **Positioning** of YP as more comfortable participating separately limited scope for more direct dialogue and balancing of **Power Relations** between different stakeholder groups.	Youth advisors’ existing relationships with the researchers, and previous involvement in sexual health research, meant that they were familiar with organisational safeguarding policies, and had actively and critically considered practices that promote open and frank discussion around sex (including consent) while respecting the need for boundaries around privacy. Acknowledging that talking about sexual consent can act as a reminder of difficult experiences, an agreement was made that a researcher would contact advisors after each meeting to ‘check in’. When co-designing research tools youth advisors encouraged researchers to identify and share additional sources of support in the event of difficult or triggering discussion. Workshops and interviews were organised through organisations that had existing links with the YP and who were tasked with checking in with YP after their participation.
**PLACE** *How does the social, physical and virtual context shape what forms of participation are possible or desirable?*	The team sought to identify physical spaces conducive to CEYP participating in discussions about access to SRH services. An initial consultation event held within the local SRH service saw no YP attend. The team reflected and consulted with YP on possible reasons for this (**Protection, Power Relations**) and moved later events to a ‘safe’ location (a youth café) familiar to YP. More could have been done to consult with YP earlier about mutually suitable locations, and to explore the possibilities of virtual social spaces in which YP could meet, extend discussion and build connections beyond the project. However, budget constraints limited options.	In order to increase feelings of safety and confidence to participate in discussions about condoms and contraception (**Protection, Power Relations**), in-person workshops were held in settings familiar to YP (eg, youth group spaces). Due to the emergence of COVID-19 and subsequent UK-wide lockdowns, remaining workshops had to be rearranged virtually at a time of rapid change in social norms and practices of digitally-mediated interaction. Attempts to create safe and inclusive digital spaces for SRH-related discussions included using digital tools (**Process**) that allowed anonymous input (eg, Menti polls) and recommending ahead of time that YP find a private space where they could not be overheard. However, the unanticipated and sudden shift to virtual workshops inhibited a fuller consideration of challenges of digital SRH-related PPI.	Researchers and young advisors recognised the importance of the physical location of meetings in making participation possible, and jointly agreed them. Discussion groups took place in organisations that YP attended and felt comfortable in (**Protection**). Although facilitating and hosting discussions with organisations offered pragmatic and safeguarding advantages, it also meant that staff acted as gatekeepers to YP’s involvement. In organisations working with YP under and over the age of 16 this led to potentially challenging conversations about the inclusion and exclusion of YP on the basis of age.
**PROCESS** *How did the methods structure and enable participatory exchange, and critical and creative thought?*	The project was informed by experience-based co-design (EBCD), an approach envisioned to enable service users and staff to co-design care pathways. Activity-based methods were used to enable CEYP’s engagement and dialogue around SRH access (eg, feedback exercises; ranking activities; voting on priorities). While these activities were designed with input from the experienced Youth Worker whose conversation with CEYP sparked the initial project idea, project timelines and a focus on identifying feasible solutions (**Purpose**) limited opportunities for YP to be involved in identifying and developing activities to enable participatory exchange and creative thought.	Various activity-based methods were used to surface YP’s views and facilitate critical exchange about the priorities, methods and recommendations of the study (eg, drawing activities; creating and voting on priorities). As different YP were involved at different stages of the study (ie, some YP were involved in multiple conversations, others participated only once), YP’s involvement was framed as an “ongoing conversation”, with concerted effort placed on summarising inputs from earlier workshops/discussions in order to put YP into conversation with one another, even when they were not physically co-present.	Involving YP in design of research tools meant that they were more engaging and accessible for the wider group of YP. For example, young advisors’ recommendation to watch and collaboratively review short films about sexual consent was very effective in stimulating discussion. Subsequently, including young advisors in sense-checking findings and meeting with the commissioners towards the end of the process resulted in a rich and creative dialogue that opened up possibilities wider than the original brief. One youth advisor stayed involved beyond the research project and contributed to a multi-year, multi-sector collaboration (including NHS health improvement staff, digital communications experts, youth workers, YP) that resulted in the development of sexual communication films for the commissioners.*

**Bold text** denotes interlinkages between the 7P domains (eg, between Purpose and Process) relevant to a specific reflection.

*These films, and details of how they were collaboratively produced, are available at: https://www.awkwardmoments.co.uk/.

CEYP, care-experienced young people; LGBTQ+, lesbian, gay, bisexual, transgender, queer and others; NHS, National Health Service; PPI, patient and public involvement; SRH, sexual and reproductive health; YP, young people.

## Results

Analysis of PPI with YP in each project is presented in table 2. Cross-project insights relating to each domain of the 7P framework are presented below.

### Purpose

The 7P model locates purpose (ie, project aim) centrally to convey that this should orient the project, and be regularly reflected on throughout. Each project was initiated by NHS sexual health decision-makers (in one case in collaboration with a youth organisation), either through professional impetus to address a particular issue (eg, recognised need to develop resources on consent), or directly informed by YP feedback about challenges accessing SRH services. Despite adult-initiation, all projects sought to actively involve YP in developing project objectives, and not solely as research participants. YP’s involvement in early-stage priority-setting discussions proved invaluable to honing (and sometimes reworking) the overarching goal and objectives of each project, although differences between YP’s priorities and those commissioning the research required careful management. Where divergent views on purpose arise, creating opportunities to build consensus and feedback loops to communicate the rationale underlying decisions taken are crucial.

### Positioning

The concept of positioning invites reflection on how cultural framings of YP shape what is considered possible in terms of their contribution within PPI. Within SRH research and policy, YP are routinely described as “experts in their own lives” who should be involved in decision-making about services and policies that affect them.^24^ The extent to which this is realised is, however, debatable. Each of our projects, for instance, aspired to position YP as co-contributors, including intentionally using language of “collaboration” to convey the status placed on their input. Yet these framings sat in tension with other aspects; for example, YP were not in leadership roles across any of the projects, and the format and timing of their contributions was largely decided by project teams. On reflection, opportunities to realise more ambitious positionings of YP were constrained by implicit caution about more equitably shared decision-making, especially in projects that necessitated relationship building with YP within limited timeframes. This raises questions about potential disconnect between intended and actual positionings of YP, especially when PPI activities are one-off. As projects progressed and trust developed, however, YP’s positioning evolved (eg, from “advisors” consulted about predefined issues to “co-creators” involved in generating policy recommendations). Creating space to talk with YP about the terminology used to describe their involvement may help surface tensions around positioning, and mitigate uneven power relations.

### Perspectives

This domain requires thinking about diversity in the voices included - and not included - within PPI, and recognising gaps between intention and reality. Our projects utilised various strategies to involve YP with different experiences and identities, including: recruiting through youth organisations supporting a diverse range of YP; co-developing research tools with YP to improve accessibility; inviting input through a variety of means aiming to appeal to YP with different interests and abilities, and intentionally working with YP often underrepresented in SRH-related PPI (eg, care-experienced YP). However, ensuring diversity of perspectives within PPI takes time and resources. Evidence indicates YP can find it easier to engage with research when approached by someone they trust, such as a youth worker or teacher.^25 26^ An inherent challenge for teams conducting PPI is building trusting, reciprocal relationships with multiple gatekeeping organisations, especially in underresourced sectors where staff turnover can be high (eg, education, third sector youth organisations) and capacity to support YP to engage with research is limited.

### Power relationships

As power is relational, the 7P model encourages a critical lens on its distribution throughout PPI. All projects worked to develop an ethos of respect, where YP felt their contributions were valued. This included providing opportunities for participation that challenged power imbalances between adults and YP (eg, involving young advisors in analysis meetings, developing multiple options for expressing ideas and opinions, and foregrounding YP’s ideas in meetings with other stakeholders). Nevertheless, differences in expertise (eg, on research methods), tight timescales and budgets, and institutional constraints can inhibit shared decision-making. A major tension exists, for instance, where professionals are required to shape the project vision and objectives to secure funding and ethical approval to work with YP, thereby constraining YP’s involvement in early decision-making. Possible strategies to mitigate uneven power dynamics within PPI include (co)developing funding proposals (ideally in collaboration with YP) that embed flexibility for projects to be taken in new directions, resource for capacity-building, and time to explicitly discuss power relationships early in PPI activities.

### Protection

The domain of protection urges reflection on vulnerabilities alongside capabilities, including ways in which PPI may be experienced as a personal, social or political risk. Within the field of SRH, these risks may feel especially acute, with YP concerned about negative repercussions of involvement in projects related to SRH – either during participation itself (eg, stigmatising responses from others in a discussion), or stemming from others’ (eg, parents, friends) knowledge of their participation. Our efforts to promote protection included collectively agreeing ground rules for group work with YP. Yet, for the most part, safety procedures were limited by being decided by researchers, approved by ethics committees, and only then enacted with YP. In contrast, the 7P model invites reflection on ways that YP can be positioned as “co-creators of safety”, again underlining the importance of YP’s early involvement in shaping PPI practices.

### Place

The 7P model conceptualises place as both physical and relational, and calls for consideration of the “exclusionary and/or inclusionary implications” of spaces in which participation occurs. Within in-person PPI activities, sensitisation to the significance of place led teams to arrange meetings in settings familiar to YP (eg, youth centres). In these venues, researchers were the “outsiders” required to find their bearings and navigate the social and logistical uncertainties of unknown environments – a strategy aiming to reduce power hierarchies, and promote feelings of ease, confidence and safety among YP. Generating such feelings in virtual spaces can be a particular challenge for SRH-related PPI, particularly where YP are concerned about securing privacy to express their views (eg, being overheard at home). Our efforts to promote YP’s comfort participating in virtual PPI included encouraging YP to consider beforehand where to situate themselves; recruiting YP already known to one another for group work; and using digital tools (eg, online polling, collaborative notepads, breakout rooms) to allow those not comfortable talking in larger groups to contribute. Nevertheless, exclusions within these spaces likely still occurred, including among those experiencing data poverty or low confidence using certain digital platforms. Some tools (eg, virtual whiteboards) did not work well via digital devices that YP often use to participate (eg, smartphones vs laptops). Such realities underline the importance of working with YP early on to think through the domain of place, including the ethical and logistical factors that enable safe participation.

### Process

Reflection on process encourages attention to alignment between PPI goals and methods. Our projects sought to operationalise our participatory ethos by using methods that facilitated critical dialogue with YP about existing sexual health services and resources, and possibilities for change. Most methods involved group-based activities – some in-person (eg, creative drawing to map factors affecting SRH service use; reviewing films about consent to prompt discussion about future resources) and some remote (eg, co-developing policy recommendations via digital notepads). While these methods generated valuable participation, emphasis on activities requiring direct interaction between YP likely excluded those uncomfortable being visible within discussions, especially regarding sexual health. Moreover, while sustained engagement throughout a project is often valorised as somehow more “meaningful”, expectations of ongoing involvement may be a barrier to those who do not wish, or are not able, to do so. In order to include diverse voices, YP need to feel safe to “dip in and out” of involvement work and know that their contributions will be valued, however short-lived. Participation is routinely framed as “an ongoing conversation” rather than a singular event.^23^ As such, envisaging conversation as a carousel, where new conversants can join the conversation while others can drop out, may be a useful way to conceptualise inclusive and ongoing dialogue within PPI.

## Discussion

If “we” (ie, the SRH community) are serious about involving young people in improving research and services, creating space for candid dialogue about the nuances, benefits and challenges of PPI is key. Frameworks of youth participation, such as the 7P model, can provide productive “thinking tools” to support these conversations. In our analysis of PPI across three projects, reflection on seven interconnected domains of participation led to new insights that will strengthen our future practice. In table 3 we identify several potential challenges for meaningfully involving YP in SRH-related PPI, and offer practical recommendations at two levels – action that can be taken within specific projects, and calls for change within the wider SRH system.

**Table 3:** Recommendations for meaningfully involving young people in sexual and reproductive health (SRH)-related patient and public involvement (PPP)

Challenge/problem	Recommendations for project level actions (eg, PPI within specific research studies and service improvement processes)	Recommendations for systems-level change
Young people’s involvement is sought too late, minimising opportunities to meaningfully shape project purpose and objectives	Build relationships with and capacity of YP to enable engagement in the early stage of projects. Involve YP early and often in agenda-setting discussions shaping the direction of research or service improvement processes.	Increase buy-in from those with decision-making power in SRH (eg, clinical leads, policymakers, commissioners) to understanding that YP’s involvement in shaping SRH services and research evidence is integral to improving sexual health. Increase commitment to, and accountability for, involving YP in SRH agenda-setting activities at local and national levels (eg, via participatory policy processes; SRH professional association activities; as lay reviewers of SRH grant applications). More resources available to involve YP in early-stage development of research grant and service improvement projects (eg, seed funding so YP can be compensated for their involvement). Flexibility from funders (including in how budgets are spent) so that funded projects with ongoing PPI can be responsive to input from YP (eg, regarding project objectives, methods, and outputs). Organisational resources invested in building capacity and confidence among SRH practitioners and researchers to plan, conduct and evaluate PPI. More resources to support SRH stakeholders to build multi-sector partnerships, share learning, and collaborate regarding PPI with YP.
Young people are not aware of how their input shapes project direction and outcomes	Prioritise feedback loops to communicate how YP’s contributions are shaping research or services. Ask YP how they want to keep in touch (eg, do not assume email is the default preference, or that YP all prefer the same communication approaches). Share project updates widely using multiple modes of communication (eg, email newsletters, blog posts, updates on social media).	
Lack of clarity about young people’s roles within PPI work and position in relation to others (eg, researchers, people running SRH services, other SRH stakeholders)	Talk with YP early on about possible roles within a project, and the terminology used to describe them and their involvement. Talk explicitly with YP about potential power dynamics (eg, between YP and project teams, between YP themselves), and collaboratively develop strategies to minimise these. Be explicit about institutional (or other) constraints on resources and capacity and how these might shape what happens in a project. Continue to be honest about these constraints and limitations throughout the project, even when that feels uncomfortable. Keep checking in on YP’s experiences of positioning and power dynamics as projects evolve.	
Lack of diversity in groups of young people involved in PPI work	Invest time in building trusting multi-sector partnerships (eg, collaboration between NHS, third sector and community organisations, academic researchers) to support diverse groups of YP to be involved, including groups often underrepresented in participatory processes relating to SRH. Consider how methods of involvement might limit participation from particular groups of YP. Do you have flexibility to offer options of how to participate? Can you ask groups who are missing from the research about whether and how they would like to be involved? Consider how location of PPI can shape involvement. For in-person activities, be mindful that institutional spaces (eg, SRH clinics, university offices) may inhibit YP’s involvement. For virtual activities, be mindful of inequities in data and WiFi access, opportunities to find private space and confidence to speak online.	
Young people may be concerned about negative repercussions of involvement in projects related to SRH	Talk with YP early on about how to create safe and inclusive spaces for PPI in relation to the particular project. Openly acknowledge potential issues around stigma and consider options for involvement that allow YP who wish to maintain privacy to still participate in a wider “conversation” (eg, options that do not rely on face-to-face discussion with other YP). Build YP’s views into developing safety protocols and institutionally-required ethics applications, ideally in collaboration with YP.	
Young people may be concerned that they are expected to give more time or commitment to PPI activities than they wish or are able	Celebrate the value of YP’s contributions, whether one-off or ongoing. Find ways to summarise previous input and create a sense of ongoing conversation between YP involved throughout the project.	
Lack of understanding of young people’s experiences of being involved in PPI, and how their participation strengthens projects, limits development of more meaningful PPI practice	Create mechanisms to regularly evaluate involvement processes so that YP can easily and safely feed back on their experiences (eg, via anonymous online polls after meetings/workshops). Integrate learning about what is (and is not) working into future practice. Create mechanisms to evaluate the impacts of involvement by documenting YP’s participation and the changes that it makes to strengthening SRH research and service improvement.	

PPI, patient and public involvement; SRH, sexual and reproductive health; YP, young people.

Key learning suggests that mainstreaming the meaningful involvement of YP within SRH service and research design requires system-wide change. In our projects, for instance, limitations arose from YP’s involvement being sought too late (ie, after projects had been conceived, funded and ethically reviewed), with limited time and resources to build trusting relationships with organisations that might support the involvement of a more diverse range of YP. Solutions to these issues extend beyond the scope of discrete projects. A systems perspective^27^ may be especially valuable for developing ways to positively disrupt current decision-making within SRH research and improvement and manifest a system where YP’s contributions are sought, valued and enacted as standard practice. What new structures and relationships are required to facilitate this, and how can these be coordinated and sustainably resourced to enhance YP’s involvement? Potential features of systems-level change might include: increased buy-in among SRH decision-makers to the understanding that YP’s involvement is integral to improving research and services; greater flexibility from funders for projects to adapt in response to YP’s contributions; and sustained investment in SRH-specific PPI communities of practice to build capacity and facilitate cross-sector collaboration among organisations supporting YP, including those often underrepresented in participatory processes.

Evaluating PPI is key to understanding what is (and is not) working, and adapting practice accordingly. As SRH services and systems seek to recover from the COVID-19 pandemic and innovate practice, we need to listen to YP and share new ideas about how to create spaces for meaningful PPI that feels safe, inclusive and keeps pace with ever-changing digital environments. It is incumbent on us to shift our practices of involvement in ways that meet YP’s needs, rather than tokenistically satisfying PPI expectations placed on us as researchers or practitioners.
