# Supplementary material for: Involving young people in sexual health research and service improvement: conceptual analysis of patient and public involvement (PPI) in three projects
Source: BMJ Sex Reprod Health. 2022 Oct 28;49(2):76–86. doi: 10.1136/bmjsrh-2022-201611 (PMC10176375; doi:10.1136/bmjsrh-2022-201611)
Supplement: Supplementary data [file bmjsrh-2022-201611supp001.pdf]

Supplementary Online File

Supplementary Figure 1: Cahill and Dadvand's 7P framework for visioning, planning, enacting and evaluating youth participation*

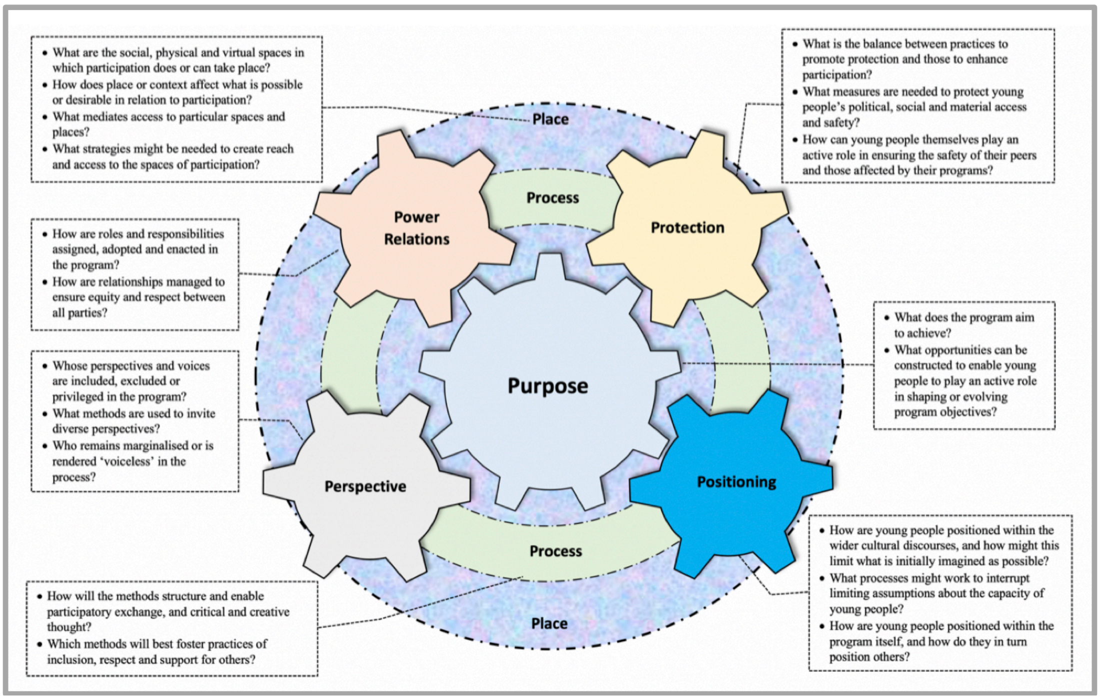

* Used with author permission

Lewis R, *et al. BMJ Sex Reprod Health* 2023; 49:77–87. doi: 10.1136/bmjsrh-2022-201611
